# Interspecific Associations of the Rare and Endangered Stingray (*Styracura schmardae*) in Caribbean Sandy‐Bottom Habitats

**DOI:** 10.1002/ece3.72471

**Published:** 2025-11-17

**Authors:** Gustavo F. de Carvalho‐Souza, Erica Sparaventi, Silvia Echeverría‐Sáenz

**Affiliations:** ^1^ Central American Institute for Studies on Toxic Substances (IRET) Universidad Nacional (UNA) Heredia Costa Rica; ^2^ Instituto de Ciencias Marinas de Andalucía (ICMAN‐CSIC) Campus Universitario Río San Pedro Cádiz Spain

**Keywords:** batoids, Caribbean Sea, commensalism, interspecific interaction, tropical sandy bottoms

## Abstract

Interspecific associations between teleost fishes and rays can enhance foraging efficiency or provide benefits, yet such interactions involving benthic stingrays remain poorly documented. Here, we report previously unrecorded associations between the rare and endangered Atlantic chupare stingray (*Styracura schmardae*) and two teleost species: the needlefish ( *Strongylura marina* ) in a nuclear–follower context (*n* = 2), and the sharksucker ( *Echeneis naucrates* ) in hitchhiking associations (*n* = 15). These records include a nuclear–follower association documented through both direct in situ observations in Costa Rica and citizen science data, and hitchhiking associations identified from a systematic review of 769 photographic records from a citizen science project, spanning multiple Caribbean locations, including The Bahamas, Belize, Colombia, Dominican Republic, Honduras, Mexico, and Panama. The observed behavior of needlefish indicates potential foraging facilitation, whereas the attachment positions of the remora imply possible hydrodynamic and feeding advantages for the hitchhiker. These findings expand understanding of the ecological role of the little‐known elasmobranch species as both a nuclear species and a host. This study also highlights the value of iEcology for revealing rare or cryptic interactions involving threatened marine species.

## Graphical abstract

We document novel interspecific associations of the endangered Atlantic chupare stingray (*Styracura schmardae*) in Caribbean sandy‐bottom habitats. Observations include nuclear–follower interactions with needlefish ( *Strongylura marina* ) and hitchhiking by sharksuckers ( *Echeneis naucrates* ), revealed through direct in situ records and citizen science data across multiple countries. These findings expand knowledge of the species' behavioral ecology and highlight the value of iEcology in detecting rare or cryptic interactions.

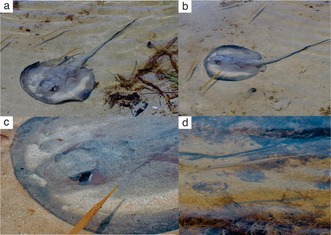

## 1 Introduction

In marine habitats, ephemeral associations are short‐lived encounters that provide immediate benefits such as enhanced foraging opportunities, protection, or predator avoidance, and can develop between a variety of species (Lukoschek and McCormick 2000; Sabino et al. 2017; Brunnschweiler 2020). Such interactions can include foraging associations, which occur when one species inadvertently benefits another (Stachowicz 2001; Pereira et al. 2013) and which are particularly relevant in dynamic environments where resource availability is spatially or temporally variable (Hines et al. 1990; McCormick 1995). Indeed, group foraging (> 2 species) has been documented to be a common behavior in reef fish communities (Auster et al. 2016). These associations can enhance prey detection, reduce predation risk, or increase foraging success, and are often observed among birds, mammals, octopuses, and fishes (Helfman 1984; Lukoschek and McCormick 2000; Sazima et al. 2007; Somaweera and Somaweera 2021).

The “nuclear–follower association” describes a commensal foraging in which one species (the nuclear) disturbs the substrate or surrounding environment while foraging, thereby exposing prey items that are opportunistically captured by another species (the follower) (e.g., Sazima et al. 2007; Krajewski 2009; Teresa et al. 2011; Pereira et al. 2013). These associations provide advantages to the followers with minimal or no cost to the nuclear species (Lukoschek and McCormick 2000; Sabino et al. 2017). Foraging interactions have been most commonly documented in freshwater and reef ecosystems, involving a wide range of taxa such as wrasses, goatfishes, groupers, and moray eels, often followed by opportunistic predators such as jacks, snappers, or characids (Sazima et al. 2007; Sabino et al. 2017; Kawasaka 2025).

Another well‐known form of interspecific association in marine ecosystems is hitchhiking, in which remoras (Echeneidae) attach to larger hosts such as elasmobranchs, marine mammals, or sea turtles using a specialized cephalic adhesive disc (Fulcher and Motta 2006; Sazima and Grossman 2006; Brunnschweiler 2020). Although this association is often regarded as mutualistic, providing hydrodynamic benefits and mobility to the remora, and ectoparasite removal to the host, it can also entail costs for the host, including increased drag, skin abrasion, and interference with movement or respiration (O'Toole 2002; Brunnschweiler and Sazima 2006).

Despite the growing recognition of these behaviors and the relevance for the trophodynamics of ecosystems, elasmobranch associations remain poorly reported (e.g., Garrone‐Neto and Carvalho 2011; Kiszka et al. 2015; Labourgade et al. 2020). Benthic rays, in particular, are potential nuclear species because of their substrate‐disturbing foraging tactics, such as suction feeding and pectoral disc undulation, which generate sediment plumes and dislodge benthic prey (Motta and Wilga 2001; White et al. 2022). Moreover, some ray species have been recorded as hosts for echeneids in hitchhiking associations (Brunnschweiler and Sazima 2006; Brunnschweiler 2020; Nicholson‐Jack et al. 2021; Castellano‐González et al. 2025). However, detailed accounts of ray–fish associations, especially those involving rare species, are lacking, likely because of the cryptic nature and low detectability of these batoid species (Garrone‐Neto and Carvalho 2011; O'Shea et al. 2021).

The Atlantic chupare stingray, *Styracura schmardae* (Werner, 1904), is a large tropical batoid distributed across the western Atlantic, ranging from the Gulf of Campeche (Mexico) to northeastern Brazil, including the Caribbean Sea and The Bahamas (O'Shea et al. 2017; Nunes and Nunes 2020; Froese and Pauly 2025). Despite its wide range, *S. schmardae* is rarely observed and currently classified as Endangered by the IUCN, primarily because of data deficiency, low encounter rates, and evidence of population decline (Dulvy et al. 2021). This species is typically associated with soft‐bottom coastal habitats, including sandy and muddy substrates, estuarine zones, and mangrove‐fringed lagoons, at depths reaching up to 30 m (O'Shea et al. 2017; Sales et al. 2020; Dulvy et al. 2021). Recent studies in The Bahamas have documented juvenile site fidelity to shallow nursery grounds (O'Shea et al. 2021) and suggest that the species may tolerate a wide range of temperatures and salinities (Palmeira‐Nunes et al. 2025; Wosnick et al. 2025). However, little is known about its ecology, including potential interspecific associations.

Here, we describe previously unreported interspecific associations between the rare and endangered stingray, *S. schmardae*, and two fish species, the needlefish, *Strongylura marina* (Walbaum, 1792) and the sharksucker, *Echeneis naucrates* (Linnaeus, 1758), in sandy bottom habitats of the Caribbean, on the basis of direct in situ behavioral observations and photographic records retrieved through a citizen science‐based search.

## 2 Material and Methods

In situ observation was conducted in shallow waters (< 0.3 m) over a sandflat connecting the Suárez River mangrove system to the adjacent coral reef in Cahuita National Park (CNP; 9.73609° N, −82.82728° W), Costa Rica. Images and video footage were obtained using both a smartphone and a Nikon D300S camera. We followed *ad libitum* and behavior sampling protocols (Altmann 1974; Bateson and Martin 2021), whereby all behaviors displayed by fishes in association with stingrays were recorded, regardless of the duration of the interaction.

To complement our direct observations, we conducted a systematic secondary data search under the iEcology framework for *S. schmardae* on the citizen science platform (iNaturalist; https://www.inaturalist.org/) in August 2025. iEcology (short for internet ecology) is an emerging research approach that uses digital data (secondary data) originally generated for other purposes (e.g., social media, online videos, citizen science platforms) to investigate ecological patterns, species behavior, and interspecific interactions (Jarić et al. 2020; Pernat et al. 2024; Novoa et al. 2025). By mining large volumes of online content, iEcology allows researchers to detect poorly documented ecological phenomena across broad spatial and temporal scales. A total of 769 photographic observations of *S. schmardae* were reviewed on the iNaturalist platform (Supporting Information 1). Each record was manually examined to document the presence of other species in the image and to identify possible associations. Duplicate entries and records of captive specimens (e.g., from aquaria or marine parks) were excluded. Identifications of *S. schmardae* were verified on the basis of diagnostic morphological characters (Stehmann et al. 1978; Carvalho et al. 2016).

For observations suggesting potential association, we contacted the original observers via the iNaturalist platform to obtain further contextual information. This included details such as the duration of the sighting, behavior of both the stingray and accompanying fish, whether the fish were actively following the stingray, and any other ecologically relevant observations not captured in the photograph. Only records with direct information or sufficient visual evidence to infer association were retained for analysis.

Finally, we conducted a bibliographic search of literature published from 1970 to August 2025, in the Web of Science and Google Scholar databases, to verify any previously reported associations involving *S. schmardae*. Using Boolean operators (AND, OR, +), we performed a structured search with the keywords: “*Styracura schmardae*,” “ *Himantura schmardae* ,” “*Dasyatis schmardae*,” “*Trygon schmardae*,” and “*Himantura schmarde*.” Subsequently, a snowballing approach (Greenhalgh and Peacock 2005) was employed to identify additional publications by screening the reference lists of retrieved sources.

## 3 Results

**FIGURE 1:**
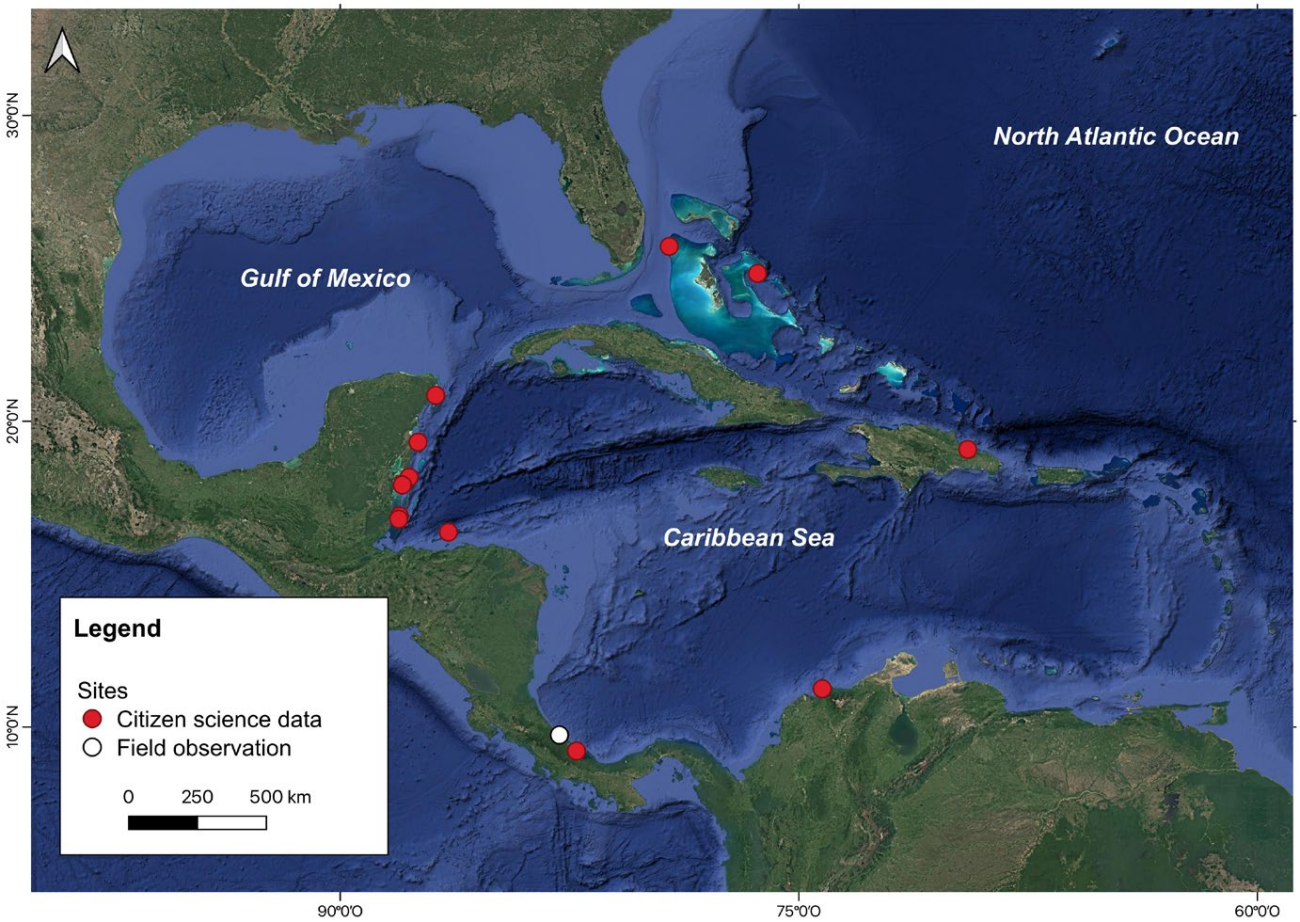
Distribution of sampling data in the Caribbean Sea. The white dot indicates records from our field observations, and the red dots represent records from citizen science.

Seventeen observations of interspecific associations between fishes and the stingray, *S. schmardae*, were recorded at various locations across the Caribbean Sea (Figure 1) through direct field observations and citizen science data (e.g., iNaturalist records; Supporting Information 1).

### 3.1 Field Observations

**FIGURE 2:**
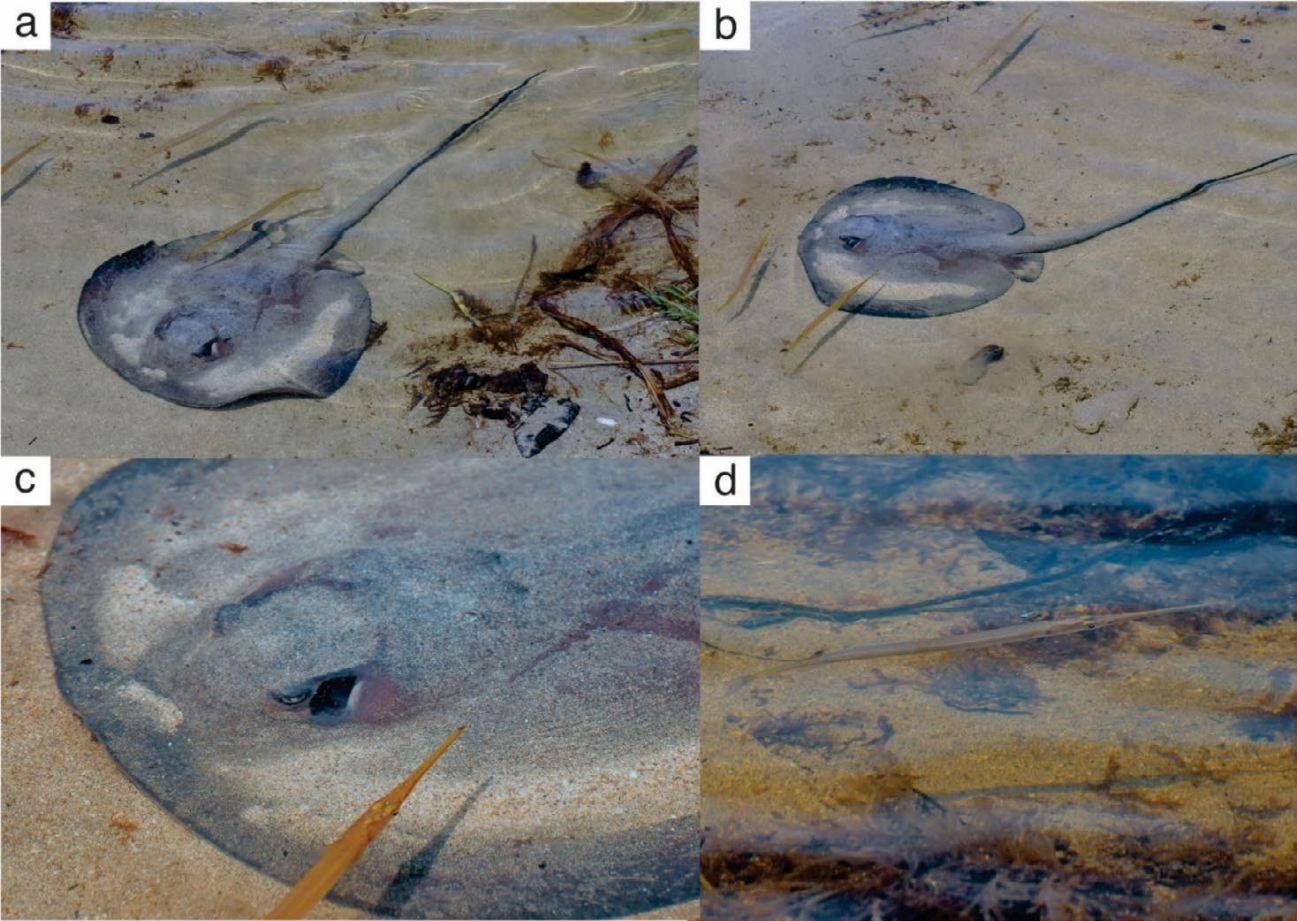
Records of needlefish ( *Strongylura marina* ) associating with *Styracura schmardae* in the Cahuita National Park, Costa Rica. (a–b) Individuals following and positioning above or ahead of the stingray; (c) *S. marina* in close proximity to the stingray; (d) *S. marina* swimming over the sandy bottom.

On 28 March 2025, we observed a foraging association between a stingray, *S. schmardae* (estimated disc width ~50 cm) and 4–5 needlefish, *S. marina* (~20 to 35 cm total length) (Figure 2a–c). The association continued for approximately 110 min, of which ~2.5 min were recorded on video (Supporting Information 2), with observations taking place between 12:50 and 14:30.

Video footage showed the needlefish maintaining close proximity to the stingray, swimming around and occasionally above or ahead of it (Figure 2c; see Supporting Information 2). Needlefish were observed at < 5 cm from the ray's dorsal surface at closest approach (Supporting Information 2) and generally remained within ~5 to 30 cm thereafter. The association persisted even during periods when the stingray paused foraging or altered its movement trajectory, with the needlefish adjusting their positions accordingly.

During active benthic foraging by the stingray, needlefish were observed inspecting the sediment plumes generated by the stingray in search of prey that were dislodged (Figure 2d). Notably, when external disturbances occurred (e.g., beachgoers approaching the area), the stingray exhibited sudden escape responses that were immediately followed by the needlefish.

### 3.2 iEcology Records

Two further nuclear–follower events were identified from iNaturalist records, in which needlefish were visibly co‐occurring in close proximity to the stingray in a nuclear–follower association (Table 1). One record was obtained at the same location (CNP) and on the same date as our direct observation, approximately 1 h earlier, likely involving the same stingray and followers, and was therefore considered part of the same field record. The other occurred in Puerto Morelos, Quintana Roo, Mexico (20.84744° N, −86.87494° W), where an observer reported 8–9 needlefish closely following two *S. schmardae* individuals throughout a ~60 min free‐diving session.

**TABLE 1:** Records of interspecific associations (nuclear–follower and hitchhiking) involving *Styracura schmardae*, including the number of associated individuals (*N*), date, site of observation, and the data source.

Species	Association	*N*	Date	Site	Source
*Strongylura marina*	Follower	4–5	28/03/2025	Cahuita National Park, Costa Rica	Present obs.
		3–4	28/03/2025	Cahuita National Park, Costa Rica	iNaturalist
		4–5	27/04/2025	Quintana Roo, Mexico	iNaturalist
*Echeneis naucrates*	Hitchhiker	1	17/06/2014	Quintana Roo, Mexico	iNaturalist
		1	05/08/2015	Islas de la Bahía, Honduras	iNaturalist
		1	15/12/2016	Stann Creek, Belize	iNaturalist
		1	11/10/2022	Hato Mayor, Dominican Republic	iNaturalist
		1	31/01/2023	Stann Creek, Belize	iNaturalist
		2	10/05/2023	Biminis, The Bahamas	iNaturalist
		1	17/12/2023	Corozal, Belize	iNaturalist
		1	29/01/2024	Corozal, Belize	iNaturalist
		1	18/07/2024	Magdalena, Colombia	iNaturalist
		1	26/07/2024	Stann Creek, Belize	iNaturalist
		1	01/10/2024	Stann Creek, Belize	iNaturalist
		1	06/02/2025	South Eleuthera, The Bahamas	iNaturalist
		1	12/03/2025	Bocas del Toro, Panama	iNaturalist
		1	16/04/2025	Mexico Rocks, Corozal, Belize	iNaturalist
		2	23/07/2025	West Bay Wall, Honduras	iNaturalist

**FIGURE 3:**
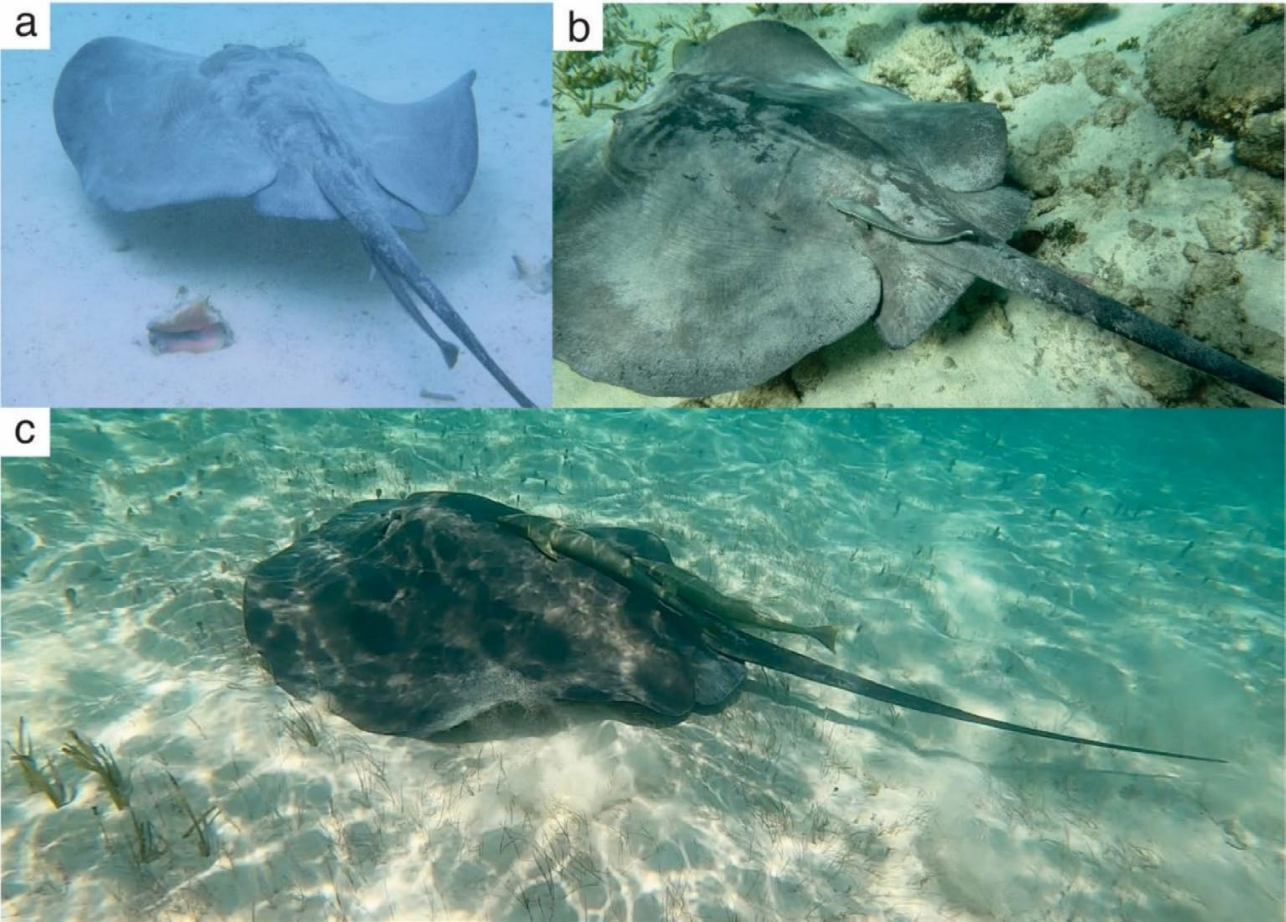
Records of sharksucker ( *Echeneis naucrates* ) hitchhiking on *Styracura schmardae* in the Caribbean. (a) Ventral side of the tail (sourced from iNaturalist, elkoster); (b) dorsal disc region (sourced from iNaturalist, tayrex); and (c) both dorsal disc region and dorsal side of the tail (sourced from iNaturalist, skarm14).

A total of 15 records of the sharksucker, *E. naucrates* , hitchhiking on *S. schmardae* were identified across the wider Caribbean (The Bahamas, Belize, Colombia, Dominican Republic, Honduras, Mexico, and Panama) (Table 1). Positions on the host varied: ventral side of the tail (*n* = 6), dorsal side of the tail (*n* = 6), and dorsal disc region (*n* = 5) (Figure 3a–c). Most records involved a single remora, but two cases documented two individuals attached to the same stingray. These observations spanned from 2014 to 2025 and included both juvenile and adult *S. schmardae*.

### 3.3 Bibliographical Review

The bibliographic search retrieved a total of 16 publications mentioning *S. schmardae* or its synonyms between 1970 and August 2025 (Supporting Information 3). Despite the species' geographic range in the western Atlantic, the review found no prior records describing behavioral associations involving *S. schmardae*. Most of the retrieved literature focused on taxonomic descriptions, distributional data, or parasitological assessments, with no documented cases of interspecific associations.

## 4 Discussion

The interspecific associations described here, involving the endangered stingray *S. schmardae*, constitute the first records of (1) a nuclear–follower association with needlefish ( *S. marina* ) and (2) a hitchhiking association with the sharksucker ( *E. naucrates* ).

Our in situ observations and image records provide baseline evidence that *S. schmardae* may function as a nuclear species facilitating foraging in shallow coastal habitats, with needlefish engaging in following behavior. The association observed fits the classical definition of a nuclear–follower association: the nuclear species disturbs the substrate while foraging, inadvertently exposing prey, which are then opportunistically captured by the follower (Sazima et al. 2007; Sabino et al. 2017). In our direct observation, needlefish remained in close proximity to the stingray throughout its movement and foraging sequences, including rapid adjustments in response to escape behavior, suggesting active spatial coordination and potential foraging benefits. Although adult needlefish are mainly piscivorous and surface‐oriented (Arceo‐Carranza et al. 2004), juveniles often consume small benthic invertebrates (Carr and Adams 1973), indicating a trophic plasticity that may facilitate opportunistic exploitation of prey dislodged by stingray foraging.

In one of the few documented nuclear–follower cases involving benthic stingrays, Kiszka et al. (2015) reported bar jacks ( *Caranx ruber* ) associating with foraging southern stingrays ( *Dasyatis americana* ) in shallow sandflat habitats in a Caribbean archipelago, with the jacks closely tracking the stingrays and striking at dislodged prey. Similar associations were also recorded in the Pacific, where trevallies followed rays such as *Taeniurops meyeni* and *Himantura fai* , often lunging into plumes created by the ray's benthic foraging activity (Kiszka et al. 2015). More recently, Arpaia et al. (2025) documented blue runner ( *Caranx crysos* ) following common stingrays ( *Dasyatis pastinaca* ), maintaining close proximity and seemingly exploiting benthic prey exposed by the ray's undulating movements, representing the first such association in the Mediterranean Sea.

In addition to nuclear–follower behavior, our study documents hitchhiking associations between *S. schmardae* and *E. naucrates* . Remoras attach to hosts to gain hydrodynamic advantages, reduce energetic costs, and access food resources, including scraps from the host's prey or ectoparasites removed from its body (O'Toole 2002; Brunnschweiler and Sazima 2006; Brunnschweiler 2020).

*E. naucrates* is a generalist hitchhiker with a remarkable diversity of recorded hosts, ranging from large predatory fishes such as the great barracuda ( *Sphyraena barracuda* ) (O'Toole 2002) to multiple elasmobranchs, including reef and pelagic sharks (e.g., *Carcharhinus* spp., *Galeocerdo cuvier* , *Sphyrna mokarran* ), manta rays (*Mobula alfredi* and *M. birostris* ), and rays (e.g., *Aetobatus narinari* ) (O'Toole 2002; Brunnschweiler and Sazima 2006; Nicholson‐Jack et al. 2021), as well as marine mammals (e.g., *Sotalia guianensis* , *Tursiops truncatus* , *Megaptera novaeangliae* ) (Fertl and Landry 1999; Wedekin et al. 2004; Souto et al. 2022) and sea turtles (e.g., *Chelonia mydas* , *Eretmochelys imbricata* , *Caretta caretta* ) (Sazima and Grossman 2006). The positions observed in our study (ventral tail, dorsal tail, and dorsal disc) are consistent with those described for other hosts, likely reflecting a balance between hydrodynamic efficiency, feeding access, and host tolerance (Brunnschweiler 2020). The broad geographic distribution of these records across the wider Caribbean, coupled with the range of host sizes (juveniles to adults), indicates that *E. naucrates* can associate with *S. schmardae* across multiple life stages and habitats.

Collectively, these observations suggest that these stingray–teleost associations may be more widespread than previously assumed, although likely underreported because of their subtle and short‐lived nature. The spatial co‐occurrence of these interactions across distant sites (Belize, Costa Rica, Mexico, and other Caribbean locations) further suggests that these associations are not a local phenomenon. Furthermore, it highlights the value of iEcology and citizen science platforms in unveiling cryptic or rare ecological interactions, especially for understudied or data‐deficient species. Similar approaches on the basis of iEcology have successfully documented diverse ecological patterns and processes, including the continental‐scale distribution of parasite infections in freshwater fishes (Happel 2019), the expansion of non‐native marine fishes in the Northeastern Atlantic and Mediterranean basins (Giovos et al. 2019; de Carvalho‐Souza et al. 2025), and the global occurrence of shark strandings (Wosnick et al. 2022).

From a conservation perspective, documenting interspecific associations such as the ones we report is fundamental for understanding the ecological role and requirements of rare elasmobranchs. *S. schmardae* remains one of the least known stingrays in the western Atlantic, and its conservation status suffers from a lack of behavioral, ecological, and distributional data (Dulvy et al. 2021). The present study documents previously unreported associations involving this species and demonstrates its ecological role as both a nuclear species and a host, while also highlighting the importance of combining traditional field observations with citizen science data as a complementary tool in the study of threatened marine fauna.

## Author Contributions

**Gustavo F. de Carvalho‐Souza:** conceptualization (lead), data curation (lead), formal analysis (lead), investigation (equal), methodology (lead), writing – original draft (lead). **Erica Sparaventi:** conceptualization (supporting), formal analysis (supporting), investigation (equal), methodology (supporting), writing – review and editing (equal). **Silvia Echeverría‐Sáenz:** conceptualization (supporting), formal analysis (supporting), investigation (equal), supervision (lead), writing – review and editing (equal).

## Ethics Statement

The authors have nothing to report.

## Conflicts of Interest

The authors declare no conflicts of interest.

## Supporting information


**Supporting Information 1.** Records of *Styracura schmardae* compiled from the iNaturalist platform, including observation ID, date, locality, coordinates (WGS84), notes on interspecific associations, number of associated individuals, and data source.

**Supporting Information 2.** Video documenting the association between the stingray *Styracura schmardae* and the needlefish *Strongylura marina* in sandy‐bottom habitat.

**Supporting Information 3.** List of publications identified in the literature review, including authors, year, title, source/journal, and data source/URL.

## Data Availability

All data supporting the findings reported in this study are included in the manuscript. The Supporting Information is publicly available.
